# Potential mechanisms for lumbar spinal stiffness change following spinal manipulative therapy: a scoping review

**DOI:** 10.1186/s12998-020-00304-x

**Published:** 2020-03-23

**Authors:** Peter Jun, Isabelle Pagé, Albert Vette, Greg Kawchuk

**Affiliations:** 1 Faculty of Rehabilitation Medicine, University of Alberta, Corbett Hall, 8205 114 Street NW, Edmonton, Alberta T6G 2G4 Canada; 2 Faculty of Rehabilitation Medicine, University of Alberta, Corbett Hall, 8205 114 Street NW, Edmonton, Alberta T6G 2G4 Canada; 3 Department of Mechanical Engineering, University of Alberta, Donadeo Innovation Centre for Engineering, 9211-116 Street, Edmonton, Alberta T6G 1H9 Canada; 4 Department of Physical Therapy, University of Alberta, Corbett Hall, 8205 114 Street NW, Edmonton, Alberta T6G 2G4 Canada

**Keywords:** Low back pain, Lumbar spine, Manual therapy, Spinal manipulation, Spinal mobilization, Spinal stiffness

## Abstract

**Introduction:**

In individuals having low back pain, the application of spinal manipulative therapy (SMT) has been shown to reduce spinal stiffness in those who report improvements in post-SMT disability. The underlying mechanism for this rapid change in stiffness is not understood presently. As clinicians and patients may benefit from a better understanding of this mechanism in terms of optimizing care delivery, the objective of this scoping review of current literature was to identify if potential mechanisms that explain this clinical response have been previously described or could be elucidated from existing data.

**Methods:**

Three literature databases were systematically searched (MEDLINE, CINAHL, and PubMed). Our search terms included subject headings and keywords relevant to SMT, spinal stiffness, lumbar spine, and mechanism. Inclusion criteria for candidate studies were publication in English, quantification of lumbar spinal stiffness before and after SMT, and publication between January 2000 and June 2019.

**Results:**

The search identified 1931 articles. Of these studies, 10 were included following the application of the inclusion criteria. From these articles, 7 themes were identified with respect to potential mechanisms described or derived from data: 1) change in muscle activity; 2) increase in mobility; 3) decrease in pain; 4) increase in pressure pain threshold; 5) change in spinal tissue behavior; 6) change in the central nervous system or reflex pathways; and 7) correction of a vertebral dysfunction.

**Conclusions:**

This scoping review identified 7 themes put forward by authors to explain changes in spinal stiffness following SMT. Unfortunately, none of the studies provided data which would support the promotion of one theme over another. As a result, this review suggests a need to develop a theoretical framework to explain rapid biomechanical changes following SMT to guide and prioritize future investigations in this important clinical area.

## Introduction

Low back pain (LBP) is the leading musculoskeletal cause of disability globally, while its financial burden continues to grow with an aging population [1, 2] . One explanation for these statistics is that, in the majority of LBP cases, the specific nociceptive source of LBP cannot be identified [1, 3]. As such, the treatment of non-specific LBP when the cause is unknown can lead to a wide variety of outcomes if interventions cannot be matched to the underlying etiology [4, 5].

Spinal manipulative therapy (SMT) is a common mechanical conservative intervention for LBP that is recommended by several clinical practice guidelines and review papers [6–8]. Recently, a series of studies with small sample size have demonstrated that spinal biomechanics of individuals with LBP could change in those who receive SMT then report improvements in disability [9–11]. In these individuals, also called SMT responders, SMT resulted in local biomechanical changes including a rapid decrease in bulk stiffness and improvement in muscle contraction. The responders also displayed increased disc diffusion post-SMT; however, the disc diffusion was measured at a different time-point to the follow-up disability measure. In comparison, these same mechanical features did not change in individuals with LBP who were non-responders to SMT. Importantly, a reduction in stiffness has been shown in two different studies [9, 10].

Taken together, these studies provide a unique investigative opportunity. Given that most interventions for LBP require weeks or months to exert their effect (e.g., exercise, surgery) [12] and few, if any, other interventions for LBP exhibit an association between self-reported and objective outcomes, this selective treatment response offers a novel starting point from which to identify a specific etiology of a subset of LBP. In addition, it may provide the opportunity to explore how a specific intervention may work to achieve this.

While the above development is unique in LBP research, it exists independently from an existing theoretical framework that would explain how the effects of SMT influence spinal stiffness and, ultimately, LBP in some, but not in other affected individuals. A theoretical framework is helpful for providing directions and priorities for future studies that would delineate the working mechanics of this phenomenon [13, 14]. This new model could provide the basis to generate valuable knowledge in understanding the LBP source and how it may be targeted with specific therapeutic interventions in the future.

With this in mind, we conducted a scoping review with the objective of identifying existing literature that would offer mechanistic explanations for a change in spinal stiffness of individuals with LBP who report improvements in disability following spinal manipulation. The results from this review could then be used to develop a theoretical framework to guide future investigations in this area.

## Methods

A scoping review is a form of review scholarship based on a framework for synthesizing available published literature on a given topic [15, 16]. A scoping review, which allows more general questioning and exploration of the literature than a traditional systematic review [16, 17], is appropriate for developing an understanding of an emerging field.

### Research question

An established scoping review methodology was used to collect and organize relevant information to examine the existing body of literature [15]. The review was guided by the following question: Are there potential mechanisms described in the literature that explain sudden changes in spinal stiffness in some, but not all individuals with LBP?

### Literature search strategy

The review process followed the recent guideline of PRISMA (Preferred Reporting Items for Systematic Reviews and Meta-Analyses) and, particularly, the extension for Scoping Reviews (PRISMA-ScR) [18]. The peer-reviewed literature was systematically searched in MEDLINE, CINAHL, and PubMed. The search terms included subject headings (MeSH terms) specific to each database and a combination of keywords relevant to SMT (including spinal manipulation and mobilization), spinal stiffness, lumbar spine, and mechanism (Additional file 1). The search period was restricted to studies published in the last two decades as the notion of spine mechanics only changing in SMT responders is less than a decade old and, thus, older studies are irrelevant for answering our research question. A manual review of articles’ references was used as an additional data source. The search strategy was reviewed with the librarian in residence at the University of Alberta (Edmonton, Alberta, Canada).

### Study selection

Eligibility criteria included: 1) publication in English; 2) quantification of lumbar spinal stiffness before and after an SMT; and 3) publication between January 1st, 2000 and June 10th, 2019 (date of the final literature search). Exclusion criteria included: 1) not using SMT as an intervention; and 2) only measuring cervical spinal stiffness. ﻿A pair of independent reviewers (PJ and IP) screened the search results in two rounds. All studies were first screened using titles and abstracts to identify relevant, possibly relevant, and irrelevant citations. Secondly, relevant and possibly relevant studies were screened in full text to determine eligibility against the above inclusion criteria. A third reviewer (GK) was involved to resolve disagreements.

### Data extraction

From the selected studies, data were extracted by 2 reviewers (PJ and IP), and any discrepancies were resolved after discussion. Extracted data from each included study contained: 1) first author and year of publication; 2) study design; 3) sample size and characteristics; 4) interventions; 5) spinal stiffness measurement and other variables/outcomes; and 6) main results.

Sentences that explained or speculated about the mechanism of SMT were manually extracted from the selected studies. Candidate sentences included wording such as “may be due / related / associated / explained / modified”, “is believed”, “was hypothesized”, “may permit / allow / result / modify / increase / decrease/ facilitate / inhibit / explained” or “have been reported / shown to”. If a sentence did not contain one of these phrases, but implied a potential mechanism underlying the effect of SMT, the sentence was also extracted. The extracted sentences were classified as either 1) a hypothesis or a re-statement from another study; or 2) a suggestion based on data from the study.

The extracted sentences were then pooled and categorized into mechanistic themes based on the context of the sentence. The sentences were analyzed within another full-text review in order to convey the original authors’ intentions; then they were grouped together with respect to anatomical references (muscles, nerves, etc.) or measurements (mobility, pain, etc.). From these groupings, themes were created by 2 reviewers (PJ and IP) after discussion.

## Results

### Overview of studies

**Fig. 1:**
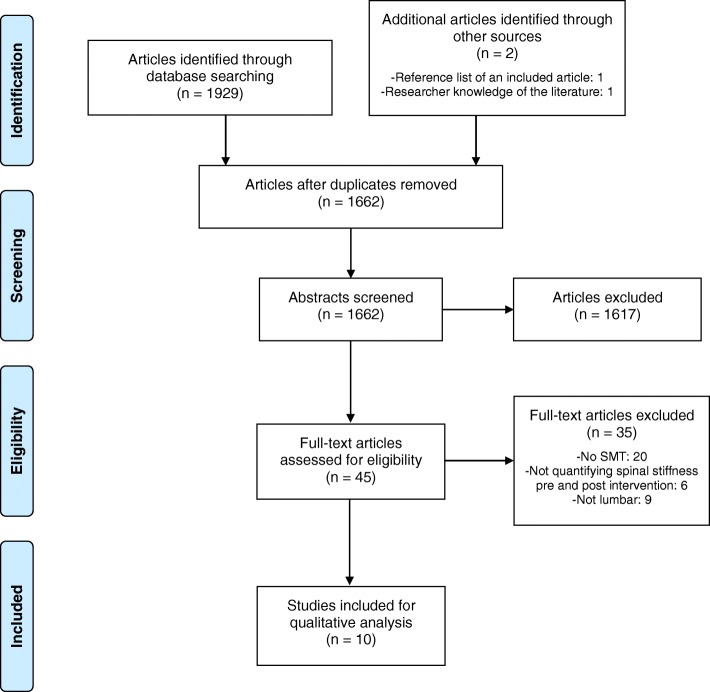
PRISMA flow chart of the scoping review

Our literature search identified 1929 studies. Two studies were manually added based on additional data sources (reviewer’s knowledge of the literature and reference list of an included study). After removing the duplicates and two rounds of screening, 10 studies met the inclusion criteria and were then processed for sentence extraction (Fig. 1).

The descriptive characteristics of the 10 included studies are presented in Table 1. Three studies exclusively recruited participants with LBP [9, 23, 26], two studies recruited healthy participants only [21, 25], and two studies recruited both participants with and without LBP [10, 20]. Finally, three of the identified studies employed animal models (conscious horses [22, 24] and anesthetized felines [19]).

**Table 1:** Descriptive characteristics of the included studies

Author (year)	Design	Sample	Interventions	Outcomes	Main results
Edgecombe et al. [19] (2015)	• A self-controlled cross-over design.	• 8 anesthetized felines.	• A VRFD device operating was used to deliver a PA SMT at four anatomic sites: SP (L6 and L7), lamina (right L6), and mammillary process (right L6). • Control intervention: contact load alone.	• The VRFD device was used to measure PA spinal stiffness at L6 SP immediately before and after each SMT application site. Stiffness at the maximal indentation load (TIS) and slope of the linear regression line best fitting the data (k) were calculated.	• ↑ in k following SMT at L6 SP compared to the control intervention. • ↓ in TIS following SMT at L6 SP and the lamina compared to the control intervention.
Wong et al. [10] (2015)	• Nonrandomized controlled study.	• 48 participants with LBP. • 59 participants with no current LBP (no intervention).	• 32 participants with LBP: A SMT consisting of the application of a PI thrust to the patient’s pelvis (both sides) in a supine position. Two treatments and one follow-up session. • 16 participants with LBP and the participants without LBP received no intervention.	• Spinal stiffness was measured by a mechanical indentation device at L3 SP with the participant in the prone position before and after the intervention and at follow-up. • Lumbar multifidus thickness ratio (LMTR) and intervertebral disc diffusion (IVDD) were also measured.	• ↓ spinal stiffness after each SMT in the SMT responders only. • ↑ in LMTR and IVDD after the first SMT in the SMT responders only. • ↓ in spinal stiffness was significantly associated with ↑ in LMTR and IVDD. • ↑ in LMTR was significantly related to the ↑ in IVDD.
Shum et al. [20] (2013)	• An experimental between-group study.	• 20 participants without LBP. • 19 participants with LBP.	• A PA MOB at the L4 involving 3 cycles of large amplitude of oscillatory PA forces (grade III). One session.	• The bending stiffness of the lumbar spine was measured by a single application of a PA load (250 N) at L4 SP by a physiotherapist. • Pain intensity, ROM and tolerance to load were also assessed.	• ↑ in curvature change, in tolerance in load application before onset of pain, and in active flexion and extension ROM following intervention in LBP. • ↓ spinal stiffness and pain intensity following intervention in LBP.
Fritz et al. [9] (2011)	• Prospective case series.	• 48 participants with LBP.	• A physical therapist or chiropractor provided a SMT using a PI thrust at the subject’s pelvis (right and left side) in supine position. Two treatments and one follow-up session.	• A mechanical indentation device was used to measure spinal stiffness (global [GS] and terminal [TS] coefficient) at L3 SP with the participant prone. Spinal stiffness was assessed before, after the intervention and at follow-up. • LM recruitment, ODI and pain intensity were also evaluated.	• ↓ in GS and TS following SMT at session 1, and in TS following SMT at session 2. • Less initial TS and greater immediate ↓ in GS were associated with greater ODI improvement. • Less initial TS was associated to greater ↑ in LM recruitment. • Being a likely SMT responder correlated with greater immediate ↑ in LM recruitment.
Stamos-Papastamos et al. [21] (2011)	• Same-subject, repeated-measures, crossover study	• 32 students without back pain.	• SMT: A rotational manipulative thrust on both sides of the L4/5 segmental level. • MOB: A 3 sets of 1 min of grade IV+ PA MOB. • Each intervention was delivered by a therapist.	• Bending stiffness was calculated by quantifying the PA force applied to L4 during 5 central PA pressures using a force platform with the participant lying prone. • Lumbar flexion and extension ROM were also measured.	• No effect of SMT and MOB on bending stiffness or ROM. • No significant correlation between Δ in bending stiffness and ROM.
Haussler et al. [22] (2010)	• Randomized clinical trial	• 24 actively ridden horses with no history of acute back problems or lameness.	• 12 horses: A single application of manually applied, HVLA, PA thrusts were applied bilaterally at T14/15, T17/18, L1/2, L3/4 and L6/S1. Once a week for 3 weeks. • 12 horses: no intervention (control group)	• Spinal stiffness was measured by the manual application of a cyclic load perpendicular to the dorsal midline over the intervertebral site of interest.	• Greater ↑ in displacement and force amplitudes during spinal stiffness assessment following SMT compared to control. • A trend of an ↑ in spinal stiffness following SMT compared to a ↓ with the control group.
Ferreira et al. [23] (2009)	• A randomised clinical trial.	• 191 participants with non-specific LBP.	• 71 participants received SMT: Joint MOB or manipulation techniques (at the discretion of the physiotherapist) were applied to the subject’s spine or pelvis. • 60 participants received general exercises. • 60 participants received motor control exercises. • Subjects attended 12 sessions over 8 weeks.	• Spinal stiffness was manually measured before and after 8 weeks of treatment by two physiotherapists using a stiffness reference device to anchor judgements of stiffness on an 11-point scale. • Pain, disability, GPE were also measured.	• ↓ mean stiffness following treatment in all groups. • No significant between group differences in Δ stiffness. • Weak correlation between Δ stiffness and Δ GPE. • Weak correlation between Δ stiffness and Δ function for the SMT group only.
Haussler et al. [24] (2007)	• A randomized crossover study	• 10 healthy adult horses.	• One HVLA PA thrusts applied to T14/15, L5/6 SPs, and left and right L1/2 articular processes using a reinforced hypothenar contact. • Control intervention: no SMT.	• Spinal stiffness was measured by manually applying a vertical force perpendicular to the dorsal midline over the intervertebral site of interest.	• ↑ in displacement and force amplitude following SMT but not following control intervention. • No change in stiffness following any intervention.
Allison et al. [25] (2001)	• A within-subjects, repeated-measures design	• 24 participants without LBP over the past 6 months.	• A standardized PA MOB (average load = 146 N, frequency = 1.5 Hz) was applied by one physiotherapist to the L3 SP for a period of two minutes.	• The SPAM apparatus was used to measure PA stiffness at L1, L3 and L5 SPs with the participant lying prone immediately before and after the intervention.	• No change in stiffness.
Goodsell et al. [26] (2000)	• A self-controlled cross-over design.	• 26 participants with LBP.	• A PA MOB manually applied (load at the discretion of the therapist) to the SP of the most symptomatic spinal level. • Control intervention: patient lying prone for 3 min.	• Spinal stiffness was measured using a device applying a PA force to the SP of the vertebra receiving the intervention with the participant lying prone before and after the intervention. • Pain and ROM were also measured.	• No difference between control and MOB on the change in spinal stiffness, overall pain and ROM. • ↓ pain on the worst movement following MOB when compared with control.

*VRFD* Variable rate force/displacement, *PA* Posteroanterior, *SMT* Spinal manipulative therapy, *SP* Spinous process, *TIS* Terminal instantaneous stiffness, *LBP* Low back pain, *LMTR* Lumbar multifidus thickness ratio, *IVDD* Intervertebral disc diffusion, *MOB* Mobilization, *ROM* Range of motion, *PI* Posteroinferior, *GS* Global stiffness, *TS* Terminal stiffness, *LM* Lumbar multifidus, *ODI* Oswestry disability index, *Δ* change in, *HVLA* High-velocity and low-amplitude, *GPE* Global perceived effect, *SPAM* Spinal posteroanterior mobilization

Importantly, the studies varied in the application of SMT. These differences included the use of spinal manipulation or mobilization at the discretion of the therapist [23], spinal manipulation and posteroanterior (PA) mobilization delivered by a clinician at L4/5 [21], lumbopelvic manipulation delivered by a clinician [9, 10], spinal manipulation delivered by a mechanical device with an external frame [19], PA mobilization delivered by a clinician at L3 [25], L4 [20], or SMT delivered to the most painful lumbar spinal level [26].

Regarding the measurement of spinal stiffness, most studies used a device with an external frame [9, 10, 19, 25, 26], two studies used a handheld device [22, 24], and two studies calculated bending stiffness during PA mobilization [20, 21]. Ferreira et al. [23] used a reference device allowing clinicians to anchor their subjective impression of spinal stiffness to a reference measurement.

Finally, SMT effects on spinal stiffness greatly varied between studies. A decrease [9, 10, 20] or no change [21, 25] in spinal stiffness following SMT were the most common results, while other studies revealed a non-statistically different change when compared with another treatment or a control intervention [22–24, 26]. Different effects depending on the SMT location have also been reported by Edgecombe et al. [19].

### Mechanisms

The reviewers were unable to identify any fully developed descriptions of mechanisms in the literature that would explain rapid changes in biomechanics following SMT. As such, we extracted sentences from the selected studies which illuminated possible mechanisms (Table 2). From these sentences, 7 mechanistic themes were identified: 1) change in muscle activity; 2) increase in mobility; 3) decrease in pain; 4) increase in pressure pain threshold; 5) change in spinal tissue behavior; 6) change in the central nervous system (CNS) or reflex pathways; and 7) correction of a vertebral dysfunction. While most studies expressed the potential mechanisms as hypotheses or re-statements from one or multiple articles, the first 5 themes were derived from sentences taken from studies that collected data.

**Table 2:** Mechanistic themes identified based on the sentences used in the selected studies

Mechanistic theme and studies	Specific sentences used in the studies	Related measurement
***Change in muscle activity***		
Wong et al. [10]^a^	*“decreases in spinal stiffness may permit increased disc diffusion and increased segmental motion enabling increased LM thickness ratios.”*	LM recruitment (thickness ratio)
Shum et al. [20]	“mechanical deformation of pain receptors of soft tissues may elicit activity of the paraspinal muscles, which will stiffen the motion segment … in people with back pain” “The decrease in spinal stiffness and the increase in spinal mobility after mobilization may also be due to the decreased muscle activity of the erector spinae”	N/A
Fritz et al. [9]^a^	*“the effectiveness of SMT could relate to a mechanical impact on spinal stiffness and subsequent neurophysiologic consequences facilitating muscle activity”* *“effects of SMT may be mediated by … enhancement in LM recruitment”*	LM recruitment (thickness ratio)
Haussler et al. [22]	“Manual therapy techniques may also … cause reflex muscle relaxation, altered motor function”	N/A
Ferreira et al. [23]	“reductions in involuntary muscle activity associated with resolution of pain may be responsible for the reductions in spinal stiffness”	N/A
Allison et al. [25]	“may be due to … muscle relaxation”	N/A
***Increase in mobility***		
Wong et al. [10]	“decreases in spinal stiffness may permit increased … segmental motion”	N/A
Shum et al. [20]^a^	*“changes in bending stiffness may be the mechanical mechanism responsible for the … improvement in spinal mobility.”*	ROM
Stamos-Papastamos et al. [21]^a^	*“some links were observed between changes in stiffness and changes in lumbar ROM”*	ROM
Haussler et al. [22]^a^	“Manual therapy techniques may also … improved spinal flexibility” “applying a mechanical thrust (i.e. SMT) caused a direct physiological increase in passive spinal mobility” *“indicative of producing a beneficial effect of increased passive spinal mobility or flexibility”*	Dorsoventral displacement
Ferreira et al. [23]	“it has been hypothesised that there is a relationship between spinal pain, reduced voluntary movement and abnormal spinal stiffness, and that restoration of normal spinal stiffness will result in a reduction of symptoms and a return of voluntary movement”	N/A
Allison et al. [25]	“Posteroanterior mobilization of the lumbar spine has been advocated as a treatment technique to restore spinal mobility on the basis that it will decrease spinal stiffness”	N/A
***Decrease in pain***		
Shum et al. [20]^a^	*“Large amplitude oscillations (grade III) may stimulate mechanoreceptors, leading to a decrease in pain”* *“changes in bending stiffness may be the mechanical mechanism responsible for the reduction in pain”*	Pain intensity (VAS)
Stamos-Papastamos et al. [21]	“It was hypothesized that a direct comparison of the 2 techniques [manipulation and mobilization] on the same asymptomatic subjects could possibly clarify the interaction of bending stiffness and ROM, without pain being present. Moreover, any changes on stiffness and ROM … could not be due to pain relief.”	N/A
Ferreira et al. [23]^a^	“it has been hypothesised that there is a relationship between spinal pain, reduced voluntary movement and abnormal spinal stiffness, and that restoration of normal spinal stiffness will result in a reduction of symptoms and a return of voluntary movement” “change in stiffness was associated with back pain” *“changes in stiffness occur as symptoms improve in patients with low back pain, and these changes are not directly due to the application of spinal manipulative therapy”*	Pain intensity (VAS)
Allison et al. [25]	“may be due to changes in symptom response”	N/A
Goodsell et al. [26]^a^	“Importantly, it has been theorized that hypomobile spinal joints may occur in association with low-back pain, and the stiffness of these joints may be altered by use of manipulative therapy.” *“the treatment did not produce a sufficient change in pain for a change in stiffness to be detected”*	Pain intensity (VAS)
***Increase in pressure pain threshold***		
Shum et al. [20]	“The reduction in pain and stiffness … may also be due to changes in the pain threshold”	N/A
Haussler et al. [22]^a^	*“SMT also increased the amplitude of applied force, indicative of increased tolerance to pressure”*	Applied force
Haussler et al. [24]^a^	*“The increases in dorsoventral vertebral mobility and the amount of applied pressure to the back after SMT”*	Applied force
***Change in spinal tissue behavior***		
Edgecombe et al. [19]	“the force applied during SMT application is believed to affect the local spinal tissues” “the observed increase in stiffness was the result of viscoelastic change caused from insufficient fluid recovery”	N/A
Wong et al. [10]^a^	“decreases in spinal stiffness may permit increased disc diffusion”	Intervertebral disc diffusion
Allison et al. [25]^a^	“Repeated loading of the spine causes creep and relaxation of spinal connective tissues, changing the resistance to the applied load. In some cases, micro-failure of tight connective tissue structures may decrease the resistance to movement and increase the range of movement in a restricted spinal segment” *“the initial displacement under load may have changed, reducing the length of the non-linear region of the force–displacement curve or resulting in small movements of the spine into more extension.”*	Stiffness measurement in multiple locations
***Change in the central nervous system or reflex pathways***		
Shum et al. [20]	“The reduction in pain and stiffness … may also be due to changes in the … and sympathetic nervous response” “the procedure may elicit activation of descending inhibitory mechanisms”	N/A
Fritz et al. [9]	“An SMT force has been shown to stimulate peripheral afferents, altering central nervous system (CNS) input, and enhancing motoneuron excitability.”	N/A
Haussler et al. [22]	“Manual therapy techniques may also stimulate peripheral joint receptors and central nervous system pathways”	N/A
Allison et al. [25]	“reflex modulation of the sensory and motor pathways … may be modified”	N/A
***Correction of a vertebral dysfunction***		
Haussler et al. [24]	“back stiffness is one of the primary clinical indicators of vertebral dysfunction”	N/A

^a^Studies which suggested potential mechanisms based on data

*Italicized* sentences were the authors’ suggestions based on data

*LM* Lumbar multifidus, *SMT* Spinal manipulative therapy, *ROM* Range of motion, *VAS* Visual analog scale

### Mechanistic themes

#### Theme 1: change in muscle activity

Six studies suggested a link between a change in muscle activity or recruitment and the change in spinal stiffness [9, 10, 20, 22, 23, 25]. These studies used different terms to refer to a change in muscle activity: change in lumbar multifidus (LM) thickness ratio [10], enhancement in LM recruitment [9], decrease in the muscle activity of the erector spinae [20], facilitation of muscle activity [9], presence of muscle relaxation [22, 25], correction of an altered motor function [22], and reduction in involuntary muscle activity [23]. Contrary to the other studies, Wong et al. [10] suggested that it is the change in muscle activity (i.e., LM thickness ratio) that is caused by a decrease in spinal stiffness and not the other way around. Two studies expressed this mechanism as an idea arising from the obtained data [9, 10]. The study by Wong et al. [10] showed that SMT responders exhibit a decrease in spinal stiffness following SMT as well as an increase in the LM thickness ratio and that these changes are negatively associated. Fritz et al. [9] showed that less initial terminal stiffness is associated with a greater increase in LM recruitment and that likely being an SMT responder is correlated with a greater immediate increase in LM recruitment.

#### Theme 2: increase in mobility

A total of 6 studies suggested that changes in spinal or bending stiffness are associated with an increase in mobility following SMT. Similar to the change in muscle activity, different terms were used to describe this mechanism: increased segmental motion [10], improved spinal mobility [20, 22, 25], changes in lumbar range of motion (ROM) [21], improved spinal flexibility [22], and return of voluntary movement [23]. Four studies implied that the change in spinal stiffness may result in an increase in mobility [10, 20, 23, 25], while two studies pointed toward the opposite; i.e., that the change in mobility causes the spinal stiffness change [21, 22]. More specifically, Shum et al. [20] observed a significant increase in lumbar ROM following SMT as well as a significant decrease in spinal stiffness. In addition, a trend in an increase in spinal stiffness and a significant increase in dorsoventral displacement were reported by Haussler et al. [22]. Finally, Stamos-Papastamos et al. [21] observed no change in spinal stiffness (as measured via bending stiffness) and no change in lumbar ROM following their intervention, but still suggested a change in mobility as a potential mechanism of the observed SMT effect.

#### Theme 3: decrease in pain

A decrease in pain was reported as a potential mechanism by 5 studies [20, 21, 23, 25, 26]. However, two studies suggested that the change in spinal or bending stiffness is responsible for the decrease in pain [20, 23], while the three others suggested that the decrease in pain is responsible for the change in spinal stiffness [21, 25, 26]. In line with this potential mechanism, Goodsell et al. [26] hypothesized that the non-significant change in spinal stiffness observed in their study was due to an insufficient change in pain following the treatment. Shum et al. [20] further suggested that the decrease in pain is due to the stimulation of mechanoreceptors during the PA mobilization or due to changes in the sympathetic nervous system. Finally, Ferreira et al. [23] suggested that the changes in spinal stiffness occur as symptoms improve in individuals with LBP, but that these changes are not directly due to the application of spinal manipulative therapy.

#### Theme 4: increase in pressure pain threshold

One study suggested that SMT generates an increase in pressure pain threshold, which would result in a decrease in spinal stiffness [20]. Moreover, the two studies involving horse models observed an increase in the amplitude of the applied force – until firm resistance at the end-range of motion in extension – following SMT which, according to the authors, would be indicative of increased tolerance to pressure [22, 24]. However, no change in spinal stiffness following SMT has been observed in those two studies.

#### Theme 5: changes in spinal tissue behavior

Edgecombe et al. [19] suggested that insufficient fluid recovery resulting in a change in the viscoelastic properties of the soft tissues would explain the increase in spinal stiffness following SMT observed in their study. In line with this, Allison et al. [25] suggested that repeated loading of the spine might cause creep and relaxation of the spinal connective tissues, which would change the resistance to the applied load and similarly the initial displacement under the applied load. Finally, Wong et al. [10] observed an increase in the intervertebral disc diffusion following SMT, but only in the participants classified as responders at follow-up. Responders also showed a decrease in lumbar spinal stiffness, which was, hence, negatively correlated with disc diffusion.

#### Theme 6: changes in the CNS or reflex pathways

Four studies hypothesized changes in the CNS or sensory and motor reflex pathways to be possible mechanisms for the changes in spinal stiffness and the decrease in pain following SMT [9, 20, 22, 25]. More specifically, Shum et al. [20] discussed potential changes in the sympathetic nervous system or the activation of descending inhibitory mechanisms. Additionally, Fritz et al. [9] suggested the facilitation of postsynaptic alpha motoneuron and cortico-motoneuron activity as well as improved cortical somatosensory integration as CNS changes that might result from SMT.

#### Theme 7: correction of a vertebral dysfunction

One study mentioned that an increase in spinal stiffness is one of the primary clinical indicators of vertebral dysfunction but did not discuss in more detail the definition of a vertebral dysfunction or how SMT would affect a vertebral dysfunction [24].

## Discussion

To our knowledge, this is the first review to identify existing literature that would offer mechanistic explanations of a change in spinal mechanics following a spinal manipulation. Our review identified 7 themes of potential mechanisms from 10 selected studies: change in muscle activity, increase in mobility, decrease in pain, change in pressure pain threshold, change in spinal tissue behavior, change in the central nervous system or reflex pathways, and correction of a vertebral dysfunction. Although many ideas have been proposed about how spinal stiffness may change as a result of SMT, they have not yet been brought together in a systematic way for evaluation. As such, our scoping review is an attempt to understand and prioritize potential mechanisms for future research in this area.

As SMT has been shown to induce a rapid change in spine biomechanics in some, but not all, individuals with LBP, we will divide our discussion into those two parts, capturing: 1) themes that may explain this response directly; and 2) themes that are best described as secondary in that they most likely arise from direct mechanisms. To make this distinction, we assume that the force imparted by SMT is experienced by a number of spinal tissues directly. These would include bone, muscle, ligament, nerve, and others. As such, the themes that describe the immediate involvement of these tissues would be considered to be primary themes.

### Primary themes

For the primary themes, we will limit our discussion to the possibility that the force imparted by SMT has a direct effect on the tissue of interest, resulting in a change in stiffness with no other systems involved.

#### Muscle

For this theme, we assume that the application of a direct force to the muscle will cause a change in the muscle that may impact post-SMT stiffness. Certainly, the application of direct force to a muscle can directly cause viscoelastic changes in the muscle that may result in stiffness alteration. The rapid application of force in SMT, however, would suggest a smaller viscoelastic effect than if the force were applied slowly [27]. When a muscle is pulled or extended rapidly as in the case of SMT, the time-dependent viscoelastic property of the muscle tissues hinders deformation and, thus, unlikely affects overall stiffness. Therefore, it is more likely that the muscle deformation may itself be the initiating event of other secondary mechanisms rather than a direct mechanism itself.

#### Spinal tissue

Similar to muscle, applying a force on bones, ligaments, tendons, and joint capsules, etc. may directly result in a change in segmental stiffness due to the viscoelastic phenomenon. However, the contribution of each of these spinal tissues to the change in spinal stiffness will differ as the load experienced by each tissue is different during SMT [28]. While all spinal tissues may experience a load during SMT, not all are likely to deform via direct application of force; i.e., bone will not deform. Of these tissues, the intervertebral disc is the largest soft tissue structure in the spine and the one most likely to affect segmental stiffness when deformed. Forceful stretching of a disc caused by SMT could increase the disc height temporarily [29], which may impact stiffness. In addition, SMT is known to affect disc diffusion in SMT responders [10, 30], and a degenerated disc alters lumbar spine segmental stiffness [31]. Assuming disc degeneration would affect its diffusion rate, we can make a connection that a change in disc diffusion following SMT may affect segmental stiffness. Given the observations from these various studies, the direct effects of SMT on the disc have more supporting evidence than other mechanisms with possible direct effects.

#### Nerve

It is unlikely that applying a force directly on the nerve would cause a direct change in spinal stiffness, although nerves can certainly experience such force directly. Nerve compression, direct pressure on a nerve, may cause pain, numbness, or muscle weakness, which may affect spinal stiffness; however, it would be unlikely that forces during SMT would achieve a magnitude that would cause this to happen and would most likely affect other tissues directly before the nerve would be impacted in this way.

### Secondary themes

#### Muscle activity

While it may be possible that the SMT force applied to the muscle can impact spinal stiffness directly, it is also possible that the nerve structures within the muscle are triggered by the deformation experienced by the muscle itself, which then initiate other downstream mechanisms. Deformation of muscle could activate paraspinal sensory neurons via stimulating muscle spindles [32, 33], which would increase muscle activity in return (e.g., increased LM recruitment), which is negatively correlated with spinal stiffness [9, 10]. The only studies which measured muscle activity among the included studies found a change in LM thickness ratio following SMT using ultrasound imaging [9, 10]. This supports the concept of muscle deformation being a secondary mechanism of stiffness change.

#### Increased mobility

An increase in mobility can only happen as a result of some change within the system occurring first. As such, this will be discounted as a primary theme. Put another way, whatever tissues are being affected, they subsequently allow a change in mobility or at least a theoretical change in mobility. Moreover, a change in spinal stiffness does not necessarily imply an increase in spinal mobility. Indeed, spinal stiffness represents the stiffness of underlying tissues throughout the application of load or, in other words, the tissue dynamics in response to the application of load [34, 35]. It is, therefore, possible to observe a change in the spinal stiffness without any change in spine mobility.

#### Pain

A decrease in pain intensity and an increase in pressure pain threshold have been reported separately in two themes, but one could consider these potential mechanisms together. Although the work by Wong et al. (2016) shows how experimental pain (increased pain intensity) changes stiffness [36], it is uncertain if this is mediated by direct irritation of the muscles or if this effect is mediated by another system (e.g., nervous system). Interestingly, the result of being able to endure more pain is associated with decreased stiffness [20, 37]. Therefore, a linkage may exist between a decrease in stiffness resulting in decreased activation of nociceptors as current evidence supports that manual therapy immediately increases the local pressure pain threshold [38] and induces other pain-related changes [39, 40].

#### Spinal tissue behavior

None of the selected studies specifically mentioned the potential secondary effect of change in spinal tissues for stiffness change. However, evidence exists that the mechanical stress, such as caused by SMT, could stimulate or hinder non-nociceptive, mechanosensitive receptive nerves in spinal tissues, including skin, tendons, ligaments, facet joints, and intervertebral disc [41–44], which could influence CNS function.

#### Nervous system

Nerves can be stimulated by the deformation of the tissue in which they reside, which then begins a cascade of other events [32]. Although changes in the CNS or reflex pathways were not as often mentioned as the other mechanistic themes, they may be occurring concurrently with others (e.g., change in muscle activity and decrease in pain). Previous studies have shown a change in different parameters related to the Hoffman reflex following SMT, suggesting an inhibition of the spinal cord motoneuron pool [45–47], which leads to more excitable motoneurons or a lower recruitment threshold, e.g., an increased LM recruitment. In addition to stimulating the nervous system through other tissues affected by SMT, there is a suggestion that SMT may alter the nervous system itself through neuroplasticity [48]. Considering the immediate change in spinal stiffness that is possible following SMT, neuroplasticity as a mechanism for this response is unlikely.

#### Correction of vertebral dysfunction

Finally, one study stated that “back stiffness is one of the primary clinical indicators of vertebral dysfunction” implying that SMT “corrects” the vertebral dysfunction in the presence of spinal stiffness change [24]. Since the authors did not describe in more detail what they meant by “vertebral dysfunction” and did not mention how it can be “identified” and “corrected”, this mechanism does not seem to be possible to be investigated.

### Future research

A limited number of included studies indicate the lack of evidence on this topic. Moreover, none of the studies used a method justifying a cause-and-effect relation between SMT and lumbar spinal stiffness change that would promote one theme over another. These results suggest the need to create a working model for these changes, which can guide and prioritize future investigations. Future studies should focus on verifying the identified potential mechanisms; the priority may be on the mechanisms associated with changes in muscle activity and spinal tissue behavior, as they were the primary mechanistic themes that could affect spinal stiffness directly. There also needs to be more effort taken to understand how other measures of spinal function (e.g. EMG) could be helpful in understanding post-SMT changes. Understanding the mechanism(s) for lumbar spinal stiffness changes following SMT could provide valuable knowledge on understanding the LBP source and how it may be targeted with specific therapeutic interventions in the future.

### Limitations

The main limitations of this scoping review are the small number of included studies and their heterogeneity. First, the interventions varied between studies, which might have influenced the effect on spinal stiffness and the other outcomes. For instance, spinal manipulation and mobilization have been shown to generate different physiological responses [49], and might therefore have different underlying mechanism(s) for changing spinal stiffness. Moreover, the varying inter-practitioner reliability of the manual intervention techniques [50] along with different measurement methods of spinal stiffness could have affected the outcomes as well. Studies were also excluded if not published in English, which may have resulted in missing relevant studies. As the earliest article within the search range was from almost 20 years ago, some proposed ideas are likely outdated.

## Conclusion

This scoping review identified 7 themes of potential mechanisms for the change in lumbar spinal stiffness following SMT: change in muscle activity, increase in mobility, decrease in pain, change in pressure pain threshold, change in spinal tissue behavior, change in the central nervous system or reflex pathways, and correction of vertebral dysfunction. Our review suggests the need to systematically investigate these proposed mechanisms as the selected studies did not provide supporting data to verify the cause-and-effect relations, and to develop a theoretical framework for future research. Our review also provides insights on which suggested mechanisms may have research priorities in order to understand the change in spinal stiffness following SMT.

## Supplementary information

**Additional file 1.** List of the search terms and its subject headings.

## Data Availability

All data generated or analyzed during this study are included in this published article [and its supplementary information files].

## Abbreviations

EMG: Electromyography
LBP: Low back pain
LM: Lumbar multifidus
PA: Posteroanterior
PI: Posteroinferior
PRISMA: Preferred reporting items for systematic reviews and meta-analyses
SMT: Spinal manipulative therapy
ROM: Range of motion
CNS: Central nervous system
VRFD: Variable rate force/displacement
HVLA: High-velocity and low-amplitude
CPR: Clinical prediction rule
ODI: Oswestry disability index
GPE: Global perceived effect
Δ: Change in
MOB: Mobilization
SP: Spinous process
SPAM: Spinal postero-anterior mobilization
