# Supplementary material for: Potential mechanisms for lumbar spinal stiffness change following spinal manipulative therapy: a scoping review
Source: Chiropr Man Therap. 2020 Mar 23;28:15. doi: 10.1186/s12998-020-00304-x (PMC7087370; doi:10.1186/s12998-020-00304-x)
Supplement: Supplementary file 1 — Additional file 1. List of the search terms and its subject headings. [file 12998_2020_304_MOESM1_ESM.docx]

**Additional file 1:** **List of the search terms and its subject headings**

| Theme | Search Terms and Subject Headings |
| --- | --- |
| Spinal Manipulative Therapy | Spinal manipulat*  Lumbar manipulat*  Manual therapy  ((manipulat* or mobiliz*) adj5 (lumbar or spine* or spinal))  Subject Headings:  Manipulation, Spinal/  Low Back Pain/  Chiropractic/  Lumbar Vertebrae/  Intervertebral Disc/ |
| Stiffness | Stiff*  Spinal stiffness  Low back stiffness  Lumbar stiffness  (change* adj5 stiffness)  biomechanic* phenomena*  kinematic*  mechanobiological phenomena*  Subject Headings:  Biomechanical Phenomena/ |
| Mechanism | Mechanism*  mechanism* of action  Biomechanic*  Anatom*  Physiolog*  Subject Headings:  Models, Biological/ |
